# Epidemiology and Antimicrobial Resistance Patterns of Urinary Tract Infections: A Cross-Sectional Study from Southwestern Saudi Arabia

**DOI:** 10.3390/medicina59081411

**Published:** 2023-08-02

**Authors:** Abdulaziz H. Alhazmi, Khalid M. Alameer, Bandar M. Abuageelah, Rena H. Alharbi, Mousa Mobarki, Shaqraa Musawi, Moayad Haddad, Abdullatif Matabi, Nabil Dhayhi

**Affiliations:** 1 Faculty of Medicine, Jazan University, Jazan 45142, Saudi Arabia; 2 Department of Medicine and Surgery, Batterjee Medical College, Aseer 62451, Saudi Arabia; 3 Faculty of Medical Applied Science, Jazan University, Jazan 45142, Saudi Arabia; 4 King Fahad Central Hospital, Ministry of Health, Jazan 45142, Saudi Arabia; m.hedad@gmail.com (M.H.);

**Keywords:** urinary tract infections (UTI), Gram-negative, antimicrobial resistance, multidrug-resistant (MDR), Jazan, Saudi Arabia

## Abstract

*Background:* Urinary tract infections (UTIs) are a prevalent form of urinary tract diseases affecting individuals of all ages and genders. In the Kingdom of Saudi Arabia (KSA), UTIs are a significant burden on the healthcare system, comprising 10% of all infections and ranking as the second leading cause of emergency department admissions. Despite this, limited research has been conducted in Saudi Arabia, particularly in Jazan Province, located in the southwestern region. *Methods:* This retrospective, cross-sectional study encompassed individuals with positive urine cultures who sought care at a tertiary hospital in Jazan between January 2022 and March 2023. A standardized data collection form was utilized to retrieve relevant information from microbiology lab test results and patients’ electronic medical records. Variables such as sex, urine sample collection date, bacterial isolates, antibiotic sensitivity, and resistance were collected using the data collection form. Data were analyzed using SPSS software, version 23.0 (IBM Corp., Armonk, NY, USA). *Results:* A total of 1082 urinary bacterial samples were isolated and identified. Females accounted for more bacterial infections (62.66%) than males (37.34%). Gram-negative bacteria represented 94% of all isolated strains. The most prevalent pathogens associated with UTIs were *Escherichia coli* (47.97%), *Klebsiella pneumoniae* (24.58%), and *Pseudomonas aeruginosa* (11.55%). Antimicrobial resistance patterns indicated the presence of extended-spectrum beta-lactamase (ESBL) (30.13%), carbapenemase-resistant *Enterobacter* (CRE) (1.94%), methicillin-resistant *Staphylococcus aureus* (MRSA) (0.74%), and vancomycin-resistant *Enterococci* (VRE) (0.18%). UTI incidence demonstrated a higher prevalence in September (13%) compared to other months in 2022. *Escherichia* coli, *Klebsiella* pneumoniae, and *Pseudomonas aeruginosa* were the predominant Gram-negative multidrug-resistant organisms (MDRO), accounting for 34.42%, 13.95%, and 1.63% of the population. *Conclusions:* The prevalence of UTIs caused by antibiotic-resistant microbes is notable in Jazan. Consistent with findings from other regions, *Escherichia coli* remains the most common causative pathogen of UTIs, displaying a seasonal pattern that warrants attention. Approximately 35% of reported cases involve MDRO, with ESBLs accounting for 30%. These results should raise concerns among healthcare officials, highlighting the necessity for further investigations into factors contributing to the circulation of MDRO in Jazan.

## 1. Introduction

Urinary tract infections (UTIs) are a prevalent form of urinary tract disease that can occur in both community and hospital settings. These infections are classified into three types based on the affected part of the urinary tract: urethritis, cystitis, and pyelonephritis [1,2]. UTIs can affect individuals of all sexes and age groups, with the incidence rate increasing with age. Among higher-risk groups, UTIs have an annual incidence ranging from 10% in the community to as high as 30% among hospitalized patients [3]. The World Health Organization recognizes UTI as a prevalent infection and a significant global health challenge in terms of morbidity, healthcare costs, and antimicrobial resistance rates [4]. UTIs are among the most widespread infectious diseases, affecting millions worldwide annually [4]. Moreover, the Centers for Disease Control and Prevention, recognizing the depth of this issue, has identified UTIs as a leading cause of healthcare-associated infections (HAIs) and a major driver of antibiotic consumption and misuse [5]. In the Asian continent, UTIs substantially burden public health, exhibiting diverse patterns of prevalence and antimicrobial resistance profiles. Extensive and systematic reviews have been devoted to studying UTIs in Asian countries, illustrating a high prevalence of UTIs (9.8%) that reached 14% in pregnant women and indicating the alarming emergence of antibiotic-resistant strains in the region [6,7].

UTIs can be categorized as simple or complicated. Simple infections occur in a healthy, unobstructed urinary system, while complicated infections involve a partially or completely blocked urinary system. Simple UTIs are more common and predictable, and the bacteria responsible for these infections are typically treatable. *Escherichia coli* is reported to account for approximately 80–90% of UTIs [6,7,8,9].

In the Kingdom of Saudi Arabia (KSA), UTIs significantly burden the healthcare system, constituting 10% of all infections and ranking as the second most common cause of emergency department admissions [1,2,3,8,9]. Women, in particular, are susceptible to UTIs, with approximately 60% estimated to experience at least one UTI in their lifetime, making them more prone to these infections compared to men [9].

Gram-negative bacteria are responsible for the majority (80–85%) of UTIs, with *Escherichia coli* remaining the most common causative agent (50–70%) [10]. Alongside *Escherichia coli*, other microorganisms such as *Klebsiella* spp., *Acinetobacter baumannii*, and *Pseudomonas aeruginosa* are frequently implicated in UTIs. Additionally, Gram-positive bacteria, including Methicillin-resistant *Staphylococcus aureus* (MRSA) and *Staphylococcus saprophyticus*, contribute to the occurrence of UTIs [11].

The excessive and inappropriate use of antibiotics is a significant factor contributing to antimicrobial resistance. The World Health Organization reports that 80% of antibiotics are used in the community, with 20–50% being administered improperly [12]. In recent years, the prevalence of multidrug-resistant organisms (MDRO), or called multidrug-resistant (MDR) bacteria, which are both pathogenic and opportunistic, has risen. MDR urinary tract infections are infections that are not susceptible to at least one antimicrobial agent from three or more antibiotics. Globally, notable MDR bacteria include Gram-negative bacteria, such as members of *Enterobacteriaceae* producing plasmid-mediated, extended-spectrum β-lactamase (ESBL), and Gram-positive bacteria, such as MRSA and vancomycin-resistant *Enterococci* (VRE). The emergence of MDR UTIs is a significant public health concern, demanding appropriate measures to prevent their spread and manage affected patients [13].

To enhance our understanding of UTIs, it is crucial to comprehensively determine the local bacterial etiology, track epidemiological changes over time, and assess susceptibility patterns. This information can guide the development of updated recommendations for the empirical treatment of UTIs. Therefore, this study aims to evaluate the epidemiology, identify the bacteria causing UTIs, and assess their antimicrobial resistance patterns among patients at a tertiary hospital in Jazan Province, Saudi Arabia.

## 2. Materials and Methods

### 2.1. Sample Size, Inclusion Criteria, and Exclusion Criteria

This retrospective, cross-sectional study was conducted at a tertiary hospital in Jazan, Saudi Arabia, between January 2022 and March 2023. The study included patients who visited the hospital and were diagnosed with UTI based on positive urine culture results. A total of 1082 patient files were assessed during the study period.

The inclusion criteria comprised patients with positive urine culture and a diagnosis of UTI across various clinical settings, including clinics, wards, emergency rooms (ER), and intensive care units (ICU) at the hospital. Patients with catheter-associated UTIs, UTIs in pregnant women, and cases with missing or incomplete data in their files were excluded from the study.

### 2.2. Data Collection

A unified and pre-designed data collection form was developed to gather information from test results and patients’ electronic medical records. Microsoft^®^ (MS) Excel (version 2023, Redmond, WA, USA) was used to collect and organize the data in the hospital registrar’s database. The collected information included the patient’s sex, date of urine sample collection, bacterial isolate, and antibiotic sensitivity/resistance. The confidentiality and accuracy of the gathered data were ensured.

### 2.3. Urine Collection and Analysis

Mid-stream urine samples were collected and processed according to the established protocols at the respective sample collection sites. The specimens were cultured on blood agar, cystine lactose electrolyte deficient agar (CLED), and MacConkey agar plates, followed by two days of incubation. The plates were examined daily to observe bacterial growth. Gram-stained smears were prepared initially for analysis. Traditional biochemical tests were employed to identify the bacteria, including the coagulase test to distinguish between *Staphylococcus aureus* and *Staphylococcus* coagulase-negative, and the oxidase test when we suspected *Pseudomonas* spp. Additionally, automated systems such as MicroScan (West Sacramento, CA, USA) and Vitek 2 (bioMérieux, Durham, NC, USA) were used for further identification and confirmation of the organisms [14].

### 2.4. Antibiotic Susceptibility Test

The antibiotic susceptibility testing was performed using the fully automated VITEK system (bioMérieux, Durham, NC, USA). The minimum inhibitory concentration (MIC) and antimicrobial susceptibility tests were determined for various antibiotics, including ampicillin (AMP), amoxicillin + clavulanic acid (AMC), amikacin (AMK), ceftriaxone (CRO), cefotaxime (CTX), ceftazidime (CAZ), ciprofloxacin (CIP), cefuroxime (CXM), cefazolin (CZO), cefepime (FEP), ertapenem (ETP), gentamicin (GEN), imipenem (IPM), levofloxacin (LVX), meropenem (MEM), nitrofurantoin (NIT), piperacillin + tazobactam (TZP), trimethoprim-sulfamethoxazole (SXT), and tobramycin (TOB). Five to ten colonies were selected from each sample, and the susceptibility results were interpreted according to the Clinical Laboratory Standards Institute (CLSI) guidelines (M100, 28th Edition, 2018). Bacterial isolates resistant to three or more antibiotics were classified as multidrug-resistant (MDR) [15].

### 2.5. Ethical Approval

The study received ethical approval from the Health Ethics Committee in Jazan, Saudi Arabia (Approval No. 2307, dated 12 January 2023). The confidentiality of the collected data was strictly maintained. This study involved a secondary analysis of routinely collected anonymized monitoring data. The research adhered to the ethical principles outlined in the Helsinki Declaration and the specific guidelines set by the National Committee of Bioethics in Saudi Arabia. The data were obtained from patient charts and/or laboratory databases as part of routine clinical procedures. Personal information and identifiable details of participants were excluded from the study.

### 2.6. Statistical Analysis

The collected data were organized and presented in tabulated form. Descriptive analyses were performed, including calculating means and generating frequency tables. IBM SPSS v.23 software (IBM Corp., Armonk, NY, USA) was used for data analysis. Univariate analysis was conducted to explore individual variables using statistical tests such as the chi-squared test for categorical variables.

## 3. Results

**Figure 1:**
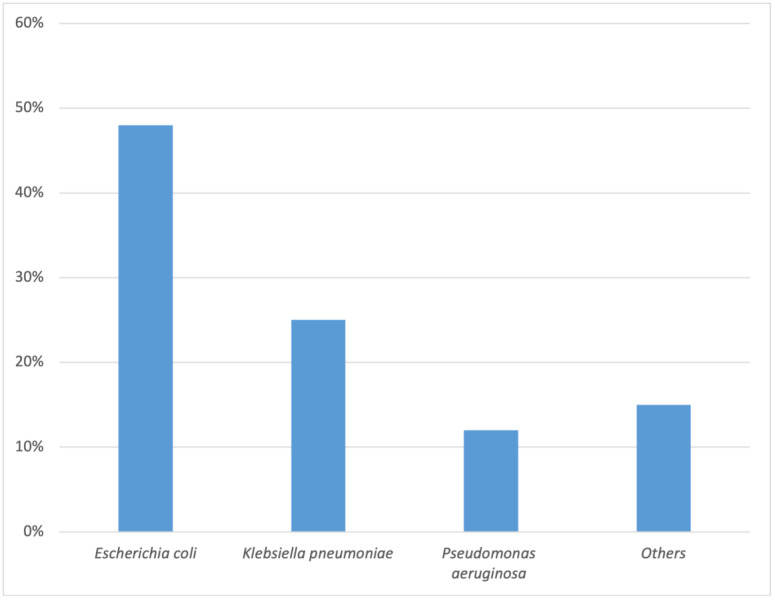
The most common reported organisms in this study.

A comprehensive analysis of 1082 urinary bacterial samples revealed several key findings. *Escherichia coli* emerged as the predominant etiologic agent responsible for community and hospital-acquired infections. The overall prevalence of bacterial infections was higher in females (62.66%) than in males (37.34%). *Escherichia coli* accounted for the majority of UTI cases (47.97%), followed by *Klebsiella pneumoniae* (24.58%) and *Pseudomonas aeruginosa* (11.55%). Other pathogens, including *Enterobacter cloacae*, *Streptococcus agalactiae*, *Enterococcus faecalis*, *Staphylococcus aureus*, *Proteus mirabilis*, and *Acinetobacter baumannii*, represented more minor cases. The distribution of uropathogens can be visualized in Figure 1.

Regarding antibiotic resistance patterns, the reported UTIs were classified into four groups. Extended-spectrum beta-lactamase (ESBL) was detected in 30.13% of cases, while carbapenem-resistant *Enterobacteriaceae* (CRE) accounted for 1.94% of cases. Methicillin-resistant *Staphylococcus aureus* (MRSA) and vancomycin-resistant *Enterococcus* (VRE) were less common, comprising 0.74% and 0.18% of cases, respectively. Detailed demographic information, antibiotic resistance patterns, and the percentage of isolated uropathogens from January 2022 to March 2023 are in Table 1.

**Figure 2:**
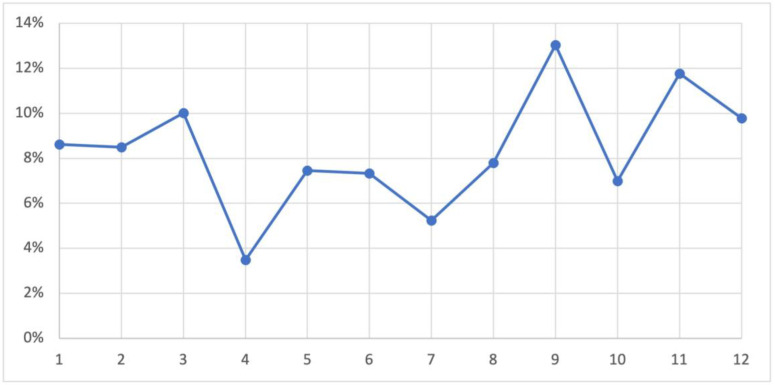
Distribution of the positive cases of UTI during the months of 2022 (*n* = 859).

The temporal distribution of UTIs throughout the study duration was reported. September came with a higher prevalence of UTIs (13%) compared to other months, while in April, the lowest prevalence was recorded, as illustrated in Figure 2.

Gram-negative bacteria constituted the majority of UTI cases (94%), while Gram-positive bacteria represented a smaller proportion (6%), as outlined in Table 2. Among 1015 identified Gram-negative bacteria, 361 cases (36%) exhibited multidrug resistance (MDR), indicating resistance to three or more tested antibiotics. On the other hand, 654 cases (64%) were susceptible to the tested antibiotics. Notably, *Escherichia coli* (34.42%), *Klebsiella pneumoniae* (13.95%), and *Pseudomonas aeruginosa* (1.63%) were the most prevalent MDR Gram-negative species. 

Table 3 provides further details on MDR infections caused by Gram-negative organisms. The analysis of gender distribution showed that 128 (12.61%) of the Gram-negative bacteria with MDRO were isolated from male patients, while 223 (21.97%) were from female patients (*p*-value = 0.498). Regarding the location of isolation, the ER had 144 (14.19%) Gram-negative bacteria with reported MDRO, followed by the wards with 86 (8.47%), ICU with 39 (3.84%), and clinics with 92 (9.06%) (*p*-value = 0.167). Considering nationality, 310 (30.54%) of the Gram-negative bacteria with reported MDRO were isolated from non-Saudi patients, while 534 (52.61%) were from Saudi patients (*p*-value = 0.096). Regarding the origin of infections, HAI showed the highest number of Gram-negative bacteria with reported MDRO, with 82 (8.08%), followed by community-acquired infections (CAI) with 183 (18.03%) (*p*-value = 0.0001). Among the Gram-negative isolates, *Escherichia coli* demonstrated the highest prevalence of MDRO, with 232 (34.42%) cases, followed by *Klebsiella pneumoniae* with 94 (13.95%), Pseudomonas aeruginosa with 11 (1.63%), and others with 12 (1.78%) (*p*-value = 0.0001).

## 4. Discussion

UTIs are a prevalent and significant health issue in the KSA, burdening the healthcare system substantially [9]. However, despite this burden, no studies have been conducted in Jazan Province, home to approximately two million people. Therefore, the aim of this study was to investigate the epidemiology of UTIs and the patterns of antimicrobial resistance in Jazan Province, located in the southwestern region of Saudi Arabia.

In this study, most UTI cases were observed in females (63%), consistent with previous studies’ findings [12,16,17]. The higher susceptibility of women to UTIs can be attributed to anatomical factors, such as the shorter urethra, as well as hormonal and behavioral factors [16,17]. Additionally, *Escherichia coli* was identified as the most prevalent causative pathogen, in line with global trends [8,17]. Other commonly isolated pathogens included *Klebsiella pneumoniae* and *Pseudomonas aeruginosa*.

Furthermore, the distribution of UTIs among various healthcare settings was reported as follows: 28.28% in the clinic, 40.94% in the ER, 9.89% in the ICU, and 20.89% in the ward. The higher proportion of UTIs observed in the ER compared to other settings indicates a significant burden in acute care settings, consistent with previous studies reporting a high prevalence of UTIs in emergency departments [18]. A seasonal pattern was also observed, with the lowest incidence in April and a higher prevalence in September. Exploring the underlying factors contributing to this seasonal variation, such as environmental changes, behavioral characteristics, or variations in healthcare-seeking behaviors due to religious seasons like Ramadan, may provide valuable insights for targeted prevention and control strategies [19].

Antimicrobial resistance is a growing concern in the management of UTIs worldwide. In this study, many UTI cases demonstrated resistance to multiple antibiotics, indicating the presence of MDROs. Notably, ESBL production was detected in 30% of the resistant isolates, accounting for a substantial portion of the resistant strains. This finding is consistent with other regional studies, although variations can be observed in western areas of the globe [8,20,21]. CRE cases were detected in a small percentage (2%), indicating the emergence of highly resistant strains. Although the prevalence of CRE in this study was relatively low compared to some other regions [22], close monitoring of the occurrence and spread of these resistant organisms is essential due to the significant challenges they pose to treatment options. These findings highlight the urgent need for effective antibiotic stewardship programs and infection control measures in the region to curb the spread of MDRO pathogens in community and healthcare settings.

We found that the predominance of Gram-negative bacteria as causative agents of UTIs aligns with global trends [3]. Gram-negative bacteria possess intrinsic resistance mechanisms, such as efflux pumps and porin mutations, contributing to their ability to resist antimicrobial agents. The high prevalence of Gram-negative MDROs, including *Escherichia coli*, *Klebsiella pneumoniae*, and *Pseudomonas aeruginosa*, further underscores the urgent need for effective screening methods to preserve the efficacy of available treatment options [23,24]. Our data revealed that most isolated resistant pathogens presented as ESBL producers, accounting for 30.13% of the cases. ESBL-producing bacteria are a significant concern as they resist a wide range of beta-lactam antibiotics, including penicillins, cephalosporins, and monobactams. This high prevalence of ESBL-producing organisms aligns with the global concern of rising antibiotic resistance, particularly among Gram-negative bacteria. Moreover, we also observed a notable proportion of CRE, a group of bacteria known for their resistance to carbapenems, considered the last resort antibiotics for severe infections. The emergence of CRE poses a significant challenge in clinical settings as it limits treatment options and increases the risk of treatment failure [3,23,24,25,26,27].

Hospital-acquired multidrug-resistant UTIs accounted for 8.08% overall, consistent with other studies that found being hospitalized is a significant risk factor for developing antimicrobial resistance [25,26]. The increased number of MDROs among patients with HAI may be attributed to several elements within the hospital environment. Firstly, prolonged hospitalization and exposure to healthcare settings can increase opportunities for cross-transmission of resistant strains among patients, contributing to the dissemination of MDROs [26]. Additionally, the prevalence of MDROs in patients with HAI may be influenced by the extensive use of broad-spectrum antibiotics, which can select for resistant strains, including ESBL-producing *Escherichia coli* and other Gram-negative bacteria. Furthermore, the compromised immune status of patients with HAI, coupled with invasive medical procedures and indwelling devices, may render them more susceptible to infections caused by MDRO. These factors could facilitate the establishment and persistence of resistant strains, leading to the higher prevalence observed in our study. A study conducted by Al-Zahrani and Alasiri in 2018 in the Asir Province, KSA, utilized multiplex-polymerase chain reaction to detect the most common carbapenemase genes. The authors identified OXA-48 as the predominant carbapenemase, followed by NDM as the second most frequently encountered carbapenemase. These findings underscore the significance of monitoring the prevalence of CRE types in the region, particularly in light of the continuous influx of visitors, especially during religious seasons. Consequently, conducting further investigations in the Jazan region is recommended to comprehensively report and trace highly resistant microbial strains, thereby augmenting our understanding of the current epidemiological landscape of antimicrobial resistance in the country [27].

In recent research, the role of intracellular bacterial communities (IBCs) has gained importance in the pathogenesis of uropathogenic infections, particularly with regard to uropathogenic *Escherichia coli* [28]. These IBCs play a significant role in promoting bacterial persistence and evading host immune responses within bladder epithelial cells, thereby contributing to the chronicity and recurrence of UTIs. Further, Ballesteros-Monrreal et al. have studied the significance of uropathogenic morphotypes, which have garnered increasing recognition in determining disease outcomes. Specifically, filamentous variants of UPEC have exhibited elevated antibiotic resistance and an enhanced ability to invade host cells [29]. Consequently, further investigation of the intricate interplay between the host, pathogen, and antibiotic therapy in UTIs, specifically in our region, is warranted. Such studies can potentially inform improved strategies for managing and preventing these recurrent and challenging infections.

While this study provides valuable insights into the epidemiology and antimicrobial resistance patterns of UTIs in Jazan Province, Saudi Arabia, several limitations exist. Firstly, it was a retrospective study conducted at a single tertiary hospital, which may limit the generalizability of the findings to the entire population of Jazan. Future studies should consider a larger sample size and a multicenter approach to provide a more comprehensive understanding of UTI epidemiology in the region. Secondly, the study focused on bacterial isolates and their resistance patterns, and further research is needed to explore other factors contributing to UTI development, such as host immune responses, virulence factors, and patient demographics. Lastly, this study only included culturable bacteria, neglecting the potential role of nonculturable bacteria in UTI cases.

Additionally, future studies should explore implementing targeted prevention strategies for UTIs, such as promoting hygiene practices, increasing awareness about risk factors, and enhancing antimicrobial stewardship programs. These studies can help reduce the incidence of UTIs and the development of multidrug-resistant strains. Moreover, conducting longitudinal studies to monitor the long-term trends of UTIs and antimicrobial resistance in Jazan Province would provide valuable insights for evaluating the effectiveness of interventions and guiding public health policies.

## 5. Conclusions

In conclusion, this study aimed to investigate the epidemiology of UTIs and antimicrobial resistance in Jazan Province, which had been understudied despite its sizable population. The study found that UTIs were more prevalent in females and primarily caused by *Escherichia coli*, in line with global patterns. ER had the highest proportion of UTI cases, indicating the substantial burden in acute care settings. Antimicrobial resistance was a growing concern, with many UTI cases demonstrating multidrug resistance, including ESBL. Effective antibiotic stewardship programs and infection control measures are crucial to combat the spread of MDROs in community and healthcare settings. However, further research with larger sample sizes and a multicenter approach is needed to enhance generalizability, explore additional factors influencing UTI development, and consider the role of nonculturable bacteria. By addressing these challenges, healthcare providers can improve patient outcomes and alleviate the burden on the healthcare system.

## Figures and Tables

**Table 1:** Descriptive analysis of the included data. (*n* = 1082).

Variable		*n*	%
Gender	Male	404	37.34
	Female	678	62.66
Location	Clinic	306	28.28
	ER	443	40.94
	ICU	107	9.89
	Ward	226	20.89
Nationality	Saudi	903	83.46
	Non-Saudi	179	16.54
Origin	ND	471	43.53
	CAI	461	42.61
	HAI	150	13.86
Alert	No alert	711	65.71
	CRE	21	1.94
	ESBL	326	30.13
	MDRO	14	1.29
	MRSA	8	0.74
	VRE	2	0.18
Gram stain	Gram-negative	1015	93.81
	Gram-positive	67	6.19
Organism	*Escherichia coli*	519	47.97
	*Klebsiella pneumoniae*	266	24.58
	*Pseudomonas aeruginosa*	125	11.55
	*Enterobacter cloacae*	28	2.59
	*Enterococcus faecalis*	16	1.48
	*Staphylococcus aureus*	15	1.39
	*Proteus mirabilis*	14	1.29
	*Streptococcus agalactiae*	20	1.85
	*Acinetobacter baumannii*	12	1.11
	Others	67	6.19

ER: emergency room. ICU: intensive care unit. ND: not determined. CAI: community-acquired infections. HAI: hospital-acquired infections. CRE: carbapenem-resistant Enterobacterales. ESBL: extended spectrum beta-lactamase. MDRO: multidrug-resistant organism. MRSA: methicillin-resistant *Staphylococcus aureus*. VRE: vancomycin-resistant *enterococci*.

**Table 2:** Variables categorized data based on Gram-stain test.

Variable		Gram-Negative Bacteria (*n* = 1015, 94%)		Gram Positive Bacteria (*n* = 67, 6%)		*p* Value
Gender	Male	374	36.85%	30	44.78%	0.195
	Female	641	63.15%	37	55.22%	
Location	Clinic	289	28.47%	17	25.37%	0.302
	ER	414	40.79%	29	43.28%	
	ICU	104	10.25%	3	4.48%	
	WARD	208	20.49%	18	26.87%	
Nationality	Saudi	844	83.15%	59	88.06%	0.395
	Non-Saudi	171	16.85%	8	11.94%	
Origin	ND	443	43.65%	28	41.79%	0.578
	CAI	429	42.27%	32	47.76%	
	HAI	143	14.09%	7	10.45%	

ER: emergency room. ICU: intensive care unit. ND: not determined. CAI: community-acquired infections. HAI: hospital-acquired infections.

**Table 3:** Gram-negative bacteria categorized based on reported MDRO.

Variable		Gram-Negative without Reported MDRO (*n* = 654, 64%)		Gram-Negative with Reported MDRO (*n* = 361, 36%)		*p*-Value
Gender	Male	236	23.25%	128	12.61%	0.498
	Female	418	41.18%	223	21.97%	
Location	Clinic	197	19.41%	92	9.06%	0.167
	ER	270	26.60%	144	14.19%	
	ICU	65	6.40%	39	3.84%	
	WARD	122	12.02%	86	8.47%	
Nationality	Saudi	534	52.61%	310	30.54%	0.096
	Non-Saudi	120	11.82%	51	5.02%	
Origin	ND	347	34.19%	96	9.46%	0.0001 *
	CAI	246	24.24%	183	18.03%	
	HAI	62	6.11%	82	8.08%	
Organism	*Escherichia coli*	287	88.04%	232	34.42%	0.0001 *
	*Klebsiella pneumoniae*	172	52.76%	94	13.95%	
	*Pseudomonas aeruginosa*	112	34.36%	11	1.63%	
	Others	16	4.91%	12	1.78%	

MDRO: multidrug-resistant organism. ER: emergency room. ICU: intensive care unit. ND: not determined. CAI: community-acquired infections. HAI: hospital-acquired infections. * Significant when the alpha criterion for *p*-value was set to 0.05.

## Data Availability

Data are available upon a reasonable request from the authors.
